# Practical Method for Isolation of Phage Deletion Mutants

**DOI:** 10.3390/mps1010006

**Published:** 2018-01-17

**Authors:** Diana Gutiérrez, Lucía Fernández, Ana Rodríguez, Pilar García

**Affiliations:** Instituto de Productos Lácteos de Asturias (IPLA-CSIC), Paseo Río Linares s/n, 33300 Villaviciosa, Spain; dianagufer@ipla.csic.es (D.G.); lucia.fernandez@ipla.csic.es (L.F.); anarguez@ipla.csic.es (A.R.)

**Keywords:** phage deletion mutant, chelating agent, temperate phage, virulent phage

## Abstract

The growing concern about multi-drug resistant pathogenic bacteria has led to a renewed interest in the study of bacteriophages as antimicrobials and as therapeutic agents against infectious diseases (phage therapy). Phages to be used for this purpose have to be subjected to in-depth genomic characterization. It is essential to ascribe specific functions to phage genes, which will give information to unravel phage biology and to ensure the lack of undesirable genes, such as virulence and antibiotic resistance genes. Here, we describe a simple protocol for the selection of phage mutants carrying random deletions along the phage genome. Theoretically, any DNA region might be removed with the only requirement that the phage particle viability remains unaffected. This technique is based on the instability of phage particles in the presence of chelating compounds. A fraction of the phage population naturally lacking DNA segments will survive the treatment. Within the context of phages as antimicrobials, this protocol is useful to select lytic variants from temperate phages. In terms of phage efficiency, virulent phages are preferred over temperate ones to remove undesirable bacteria. This protocol has been used to obtain gene mutations that are involved in the lysogenic cycle of phages infecting Gram-positive bacteria (*Staphylococcus* and *Lactobacillus*).

## 1. Introduction

The isolation and characterization of phage deletion mutants is a widely used tool for the analysis of gene function and regulation. In this regard, homologous recombination using a removable plasmid is a common technique to delete or exchange a target genome sequence in temperate phages. The recombination process is performed while the phage is inserted in the host bacterial genome as a prophage, and then, the induction of the lytic cycle by an external signal will release the mutated viral particles. However, methods to select mutant particles of virulent phages are scarce, and most of them involve the use of UV radiation or chemicals [1,2]. More recently, molecular biology techniques based on CRISPR-Cas (clustered, regularly interspaced, short palindromic repeats technology for generating RNA-guided nucleases with customizable specificities) systems have allowed accurately removing and swapping DNA fragments of some virulent phages [3,4,5,6]. One main disadvantage of these techniques is the need of previous knowledge regarding the phage genome sequence, making these methodologies time-consuming. In addition, the complete protocol is not available for all bacterial species and their phages. 

In this article, we report an easy and rapid way to isolate phage deletion mutants based on the instability of phage particles in the presence of chelating agents, such as EDTA (Ethylenediaminetetraacetic acid), sodium citrate, or sodium pyrophosphate. Chelating agents can react with the cations attached to the phage structure, breaking the bonds that maintain particle conformation [7]. Moreover, this destabilizing effect increases with temperature [8]. However, a small percentage of bacteriophages within a large population may have spontaneously lost genome fragments, which would lessen the pressure inside the head structure, and, consequently, make the mutant viral particles more stable in the presence of the chelating agent. Early work using chelating agents to isolate deletion mutants was performed in wild-type stocks of phage λ [9].

The mutations that can be selected with this method are determined by two main requirements. On the one hand, the size of the selected deletions is determined by the minimum and maximum genome size that can be packed into the phage head. Thus, very large deletions would not be selected due to the low stability of the head when the genome size is too small. On the other hand, deletions can only affect non-essential genes, as mutant selection is performed by plating, which requires the successful completion of the lytic cycle. Moreover, the phenotype of the selected phage can in some cases be screened directly after plating. For instance, mutations in the lysogeny module leading to the loss of lysogenic capability can be easily identified by the “clear lysis plaque” phenotype. Similarly, deletion of genes that regulate burst size can be recognized by the presence of smaller lysis plaques.

The procedure for selection of phage deletion mutants has been previously used to identify the repressor-encoding gene (*cI*) of the *Lactobacillus casei* bacteriophage A2. In this case, several phage deletion mutants were selected and analyzed. Deletions ranged from 0.5 to 3.5 kb and were all located in one of two genomic regions, comprising up to 7.9 kb, which were dispensable for lytic development. Those phages carrying deletions in the lysogeny module showed a “clear lysis plaque” phenotype and were unable to lysogenize their host [10]. Similarly, this method was useful to select lytic derivative mutants from the *Staphylococcus aureus* temperate phages phiH5 and phiA72. Indeed, lytic phages phiIPLA88 and phiIPLA35 (derived from phiH5 and phiA72, respectively) could only follow a lytic life cycle and turned out to be more efficient at removing *S. aureus* than their respective parent phages [11]. Further analysis revealed that point mutations in genes that were involved in lysogeny control explained their strictly lytic behavior. Thus, the repressor protein of phiIPLA35 lacks 25 amino acids at the C-terminus, while the start codon was lost in the phiIPLA88 repressor-encoding gene [12].

The protocol described here allows for the selection of phages carrying random DNA deletions, and, in addition, the subsequent isolation of those mutations affecting the lysis-lysogeny functions. This protocol was tested on *S. aureus* phages phiH5 and phiA72, but it can be applied to other phages infecting Gram-positive bacteria. In a phage therapy context, the genomic characterization of bacteriophages as well as the use of lytic phages is encouraged. Therefore, this straightforward methodology allows for the selection of lytic variants from temperate phages in a very short period of time.

## 2. Experimental Design

**Figure 1:**
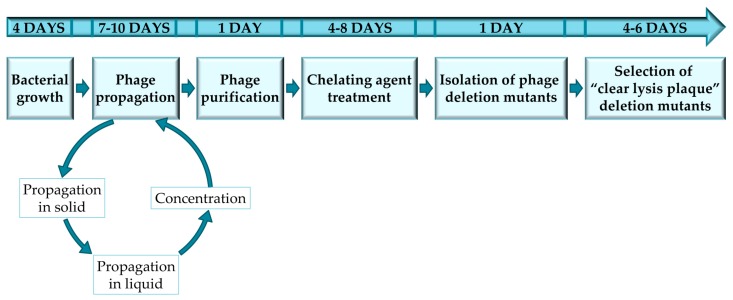
Experimental design to complete every stage of the protocol for obtaining and selecting “clear lysis plaque” phage deletion mutants.

The protocol described here has been optimized for the selection of staphylococcal phage mutants with a “clear lysis plaque” phenotype, by using sodium pyrophosphate as a chelating agent. Thus, all the protocols, materials and methods are reliable for temperate phages infecting staphylococcal species; nevertheless, this protocol was also used in temperate bacteriophages infecting *Lactobacillus* [10]. However, it must be noted that the protocol may have to be adapted for each specific bacterium-phage pair in order to ensure successful results (Figure 1). Furthermore, this protocol can also be used for the selection of mutant variants of virulent phages in which phage viability is not compromised. To note, all the steps performed in this protocol should be perform under sterile conditions using autoclaved material and a laminar airflow cabinet.

Before starting the protocol, it is necessary to determine the best conditions for growing the host bacterial culture. To do that, it is recommended to perform a growth curve at 37 °C (or optimum temperature for the phage host bacterium) in a shaker incubator during 8 h, measuring at different sampling times the absorbance, or optical density at 600 nm (OD_600_) in an “Eppendorf Biophotometer” or similar, and the bacterial concentration (CFU/mL) by plating onto solid medium plates and further incubation. For this study, the temperate phage phiH5 and the host strain *S. aureus* Sa9 were selected; although any other phage susceptible strain could be used for this purpose.

The following steps consist of propagation and purification of the phage on the selected host strain. Although these steps are not mandatory, it is advisable to purify the phage before obtaining the phage mutants, in order to ensure the success of the subsequent sodium pyrophosphate treatment. Thus, the presence of contaminants in the phage stock might entrap the sodium pyrophosphate molecules and decrease their activity, requiring a higher concentration of the compound to achieve the same effect. Phage propagation should be performed in two steps: first, in small volumes using the double-layer technique, followed by an up-scaled propagation in 1 liter of liquid medium. Prior to purification, the bacteriophage lysate should be filtered and concentrated by precipitation with NaCl and PEG 8000. Then, purification should be carried out by a conventional CsCl gradient using a ultracentrifuge. As mentioned previously, phage purification is recommended, but is not essential. However, skipping this step may lead to a lower efficiency of the protocol.

The chelating agent treatment is performed using a purified and concentrated phage stock and different concentrations of sodium pyrophosphate. Exposure of the phage to the chelating agent for 30 min at 37 °C will cause a dramatic decline in phage survival (ideally phage titers should decrease between 90 and 99% after treatment). From this treatment, phages were selected for the following rounds of treatment until obtaining a phage survival rate of 100%, even at high concentrations of the chelating agent. The concentrations of sodium pyrophosphate may vary depending on the specific phage.

After reaching a survival rate of 100%, the putative phage deletion mutants are isolated and propagated for further studies that are intended to characterize the mutations. Characterization is often carried out by DNA restriction analysis or PCR. For phages with a known genome sequence, PCR reactions can be designed to amplify regions along the genome and easily identify the deletion. For phages with an unknown genome sequence, DNA deletions should be identified by DNA extraction and further restriction analysis. This method will also provide an estimate of the deletion size.

Moreover, the selection of “clear lysis plaque” phage deletion mutants is also performed by collecting the surviving phages, isolating single lysis plaques, and testing their ability to produce turbid or transparent halos by the drop assay. Surviving bacteria inside the halo will be finally tested for susceptibility to the phage. 

### 2.1. Materials

- *S. aureus* Sa9 [11], isolated from a mastitic milk sample was used as the host strain of the phage.
- Phage phiH5, isolated from raw milk was used to select “clear lysis plaques” deletion mutants [11]
- TSB, Tryptic Soy Broth (Scharlau, Barcelona, Spain; Cat. no.: 02-200-500).
- Bacteriological agar (Cat. no.: A6686), Tris HCl (Cat. no.: 10812846001), MgSO_4_ (Cat. no.: M2643), CaCl_2_ (Cat. no.: C5670), NaCl (Cat. no.: S5886), KCl (Cat. no.: P9541), Na_2_HPO_4_ (Cat. no.: RES20908-A7), KH_2_PO_4_ (Cat. no.: NIST200B), Sodium pyrophosphate (Cat. no.: P8135), PEG 8000 (Cat. no.: 89510), CsCl (Cat. no.: 203025) and RNAse (Cat. no.: R6513) where purchased at Sigma-Aldrich, now Merck, Darmstadt, Germany.

### 2.2. Equipment

- Incubator shaker model Excella E24 (New-Brunswick Scientific UK Ltd, Cambridge, UK).
- Sanyo MIR-153 refrigerated Incubator (Sanyo electric, Ora-Gun, Japan).
- Spectofotometer BioPhotometer Eppendorf (Eppendorf Ibérica S.L.U., Madrid, Spain).
- Laminar Airflow Cabinet FASTER TWO 30 Cabinet (Manson Technology, Dublin, Ireland).
- STUART Mini Gyro Rocker SSM3 (Cole-Parmer, Stone, UK).
- Microcentrifuge 5415 R Epperdorf (Eppendorf Ibérica S.L.U., Madrid, Spain).
- Beckman Optima MAX Ultracentrifuge (Beckman Coulter, Brea, CA, USA).
- Centrifuge Thermo Scientific™ Sorvall™ RC 6 Plus Centrifuge (Fisher Scientific, Loughborough, UK).
- Vortex ZX3 (VELP SCIENTIFICA, Usmate Velate, Italy).
- Vacuum pump Diaphragm pumps, VACUUBRAND (VACUUBRAND, GMBH, Wertheim, Germany). 0.45 µm cellulose acetate filter (Whatman™, GE Healthcare, Amersham, UK; Cat. no.: 462100).
- 0.2 µm polyethersulphone filter (VWR, Radnor, USA; Cat. no.: 28145-501).
- 0.025 µm VSWP MF-Millipore™ Membrane Filters (Merck, Millipore, Cork, Ireland; Cat. no.: VSWP02500).
- 1 L, 0.45 µm pore size, Corning^®^ bottle-top vacuum filter system (Sigma-Aldrich, now Merck, Darmstadt, Germany; Cat. no.: CLS430516).
- 1.5–3 mL VWR^®^ Two-Sided Disposable Plastic Cuvette (VWR, Radnor, USA; Cat. no.: 97000-586).
- 90 mm Polystyrene Petri dishes (Labbox, Labware S.L., Barcelona, Spain; Cat. no.: PDIP-09N-500).
- Tube, thin wall, Ultra-clear, 5 mL, 13 × 51 mm ultracentrifuge tubes (Beckman Coulter, Brea, CA, USA; Cat. no.: 344057).
- PIPETMAN L Pipettes: P20L, P200L, and P1000L (Gilson, Dunstable, UK).
- Sterile aerosol filter pipet tips (VWR, Radnor, PA, USA).

## 3. Procedure

### 3.1. Bacterial Growth Conditions and Phage Titration. 4 Days

- Streak the *S. aureus* host strain onto a TSA (TSB supplemented with 2% *w*/*v* agar) plate using a sterile tip and incubate the plate at 37 °C for 18 h to obtain isolated colonies.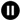 **PAUSE STEP** After the colonies are grown, plates can be stored at 4 °C for up to 1 month.
- Inoculate one *S. aureus* single colony from the TSA plate into a sterile 10 mL tube containing 5 mL of sterile TSB). Incubate at 37 °C with shaking (250 rpm) for 18 h to obtain an overnight (o/n) culture.
  **OPTIONAL STEP** Take 1 mL of the o/n culture and add glycerol to a final concentration of 20% in order to prepare a frozen stock of the host strain. Store this stock at −80 °C.
- In order to perform a bacterial growth curve, inoculate 50 mL of TSB with 1% (*v*/*v*) of the *S. aureus* o/n culture into a 100 mL sterile bottle and incubate at 37 °C with shaking.
- Take 2 mL samples at time points during incubation (0, 1, 2, 3, 4, 5, 6, 7, 8 and 24 h). Transfer 1 mL sample to a plastic cuvette and measure the optical density at an absorbance wavelength of 600 nm (OD_600_). At the same time, perform 1:10 serial dilutions of the sample into PBS buffer. Add 100 µL of the desired dilution to a TSA plate and spread using a sterile L-shape spreader until liquid dries. Incubate the plate at 37 °C for 18 h.
- Count the number of colonies in the plate containing 30–300 colony forming units (CFU). Calculate the viable cell counts of the culture using the equation:
  $$\frac{\mathrm{CFU}}{\mathrm{mL}}\;\;=\;N\;\times \;\frac{1}{DF}\;\times \;\frac{1}{V}\;,$$
  where *N* is the number of colonies counted on the plate (expressed as CFU); *DF* is the dilution factor and *V* is the sample volume poured onto the plate (expressed in mL).
- Repeat the experiment two extra times to obtain three biological replicates.
- Represent graphically the mean ± standard deviation of the CFU/mL in a logarithmic scale or the OD_600_ in the *y*-axis and the time of incubation of the culture in the *x*-axis.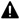 **CRITICAL STEP** The number of CFU/mL should be calculated for each host strain, but generally speaking, it is very similar among strains belonging to the same species. The calculation of the CFU/mL in the exponential culture (Cb) is necessary to allow for the accurate determination of the multiplicity of infection (MOI). Calculation of this parameter is decisive in order to achieve an optimum yield in the phage propagation procedure.

### 3.2. Phage Titration and Propagation in Small Volume. 2–5 Days

- Inoculate one *S. aureus* single colony to obtain an o/n culture (see above).
- Make 1:10 serial dilutions of the initial phage stock.
- Determine the number of infective phage particles by using the double-layer plaque assay. Take 100 µL of a *S. aureus* o/n culture and mix with 100 µL of the desired phage dilution into a 15 mL tube. Add 3 mL of semisolid TSA (0.7% agar) and pour the mixture onto a TSA plate. Let the soft agar medium solidify, and then, incubate the plate at 37 °C for 18 h. This experiment must be performed in triplicate.
- After incubation, count the number of plaque forming units (PFU) in those plates where the number is between 30–300 PFU. Calculate the phage titer of the original phage sample by using the equation:
  $$\frac{\mathrm{PFU}}{\mathrm{mL}}\;\;=\;N\;\times \;\frac{1}{DF}\;\times \;\frac{1}{V}\;,$$
  where *N* is the number of plaques of lysis counted on the plate (expressed as PFU); *DF* is the dilution factor and *V* is the sample volume poured onto the plate (expressed in mL).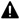 **CRITICAL STEP** If the titer of the phage suspension is ≥10^9^ PFU/mL, then the phage propagation and concentration protocol can be followed from Section 3.3. If not, propagation in solid medium should be performed before proceeding to the next step.
- In order to carry out the propagation in solid medium, take 100 µL of diluted phage stock mix with 100 µL of a *S. aureus* o/n culture into a 15 mL tube. Add 3 mL of TSA 0.7% and pour the mixture onto a TSA plate. Repeat the procedure to get 5 plates. Let the medium solidify and then, incubate the plates at 37 °C for 18 h.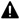 **CRITICAL STEP** After incubation, the overlaid plate should be completely transparent reflecting the occurrence of confluent lysis. The minimum concentration of phages that should be plated to ensure confluent lysis varies between 10^4^ and 10^6^ PFU/plate. Add 1 mL of SM buffer to each plate and incubate with gentle shaking (60 rpm) at 20 °C for 1 h. Recover the SM buffer from the five plates and transfer it to a 15 mL tube. Centrifuge 10 min at 13,600 × *g*. Transfer the supernatant containing the phages to a new sterile tube and perform phage titration again. If it is needed, solid propagation can be repeated again with this new phage stock in order to obtain a concentration ≥10^9^ PFU/mL.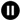 **PAUSE STEP** Phage stock solutions can be kept at 4 °C for up to three months after filtration using a 0.45 µm cellulose acetate membrane filter. For longer periods of time, a phage stock can be kept at −80 °C with 20% of glycerol.

### 3.3. Phage Propagation in Large Volume and Concentration. 5 Days

- Inoculate one *S. aureus* single colony from the TSA plate into a sterile 10 mL tube containing 5 mL of sterile TSB. Incubate at 37 °C with shaking (250 rpm) for 18 h to obtain an o/n culture.
- Inoculate one sterile 100 mL bottle containing 50 mL of sterile TSB with 1% *v*/*v* of the *S. aureus* o/n culture and incubate at 37 °C with shaking.
  **OPTIONAL STEP** Supplement the growth medium with 10 mM CaCl_2_ and 10 mM MgSO_4_. Addition of these cations is recommended for most phages, although it is not always strictly necessary, as some phages can be propagated efficiently without them.
- Take 1 mL sample after 1 h incubation and measure the OD_600_. Continue incubating the culture at 37 °C with shaking until it reaches the exponential growth phase.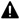 **CRITICAL STEP** The bacterial cells should be in the exponential growing state to ensure proper phage propagation.
- Transfer the exponential growing bacteria to four sterile 50 mL tubes (10 mL in each tube). One tube will be the control and the other three tubes will be used for phage propagation with three different MOIs (0.1, 1 and 10). To calculate the volume (V_0_) of the phage that should be added to the different tubes follow the equation:
  $$V_{0}\;=\;\frac{V_{1}\times C_{1}}{C_{0}}\;,$$
  where *V*_1_ is the final volume of the mixture (in this case 10 mL); *C*_0_ is the concentration of the previously titrated phage stock (≥10^9^ PFU/mL), and *C*_1_ is the desired final phage concentration. For calculating *C*_1_ follow the equation:
  $$C_{1}\;=\;C_{b}\;\times MOI\;,$$
  where *C_b_* is the concentration of the bacterial culture at an exponential growing phase (expressed as CFU/mL) and MOI is the multiplicity of infection defined as PFU/CFU.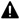 **CRITICAL STEP** The optimal MOI for phage propagation should be determined by testing different MOIs (MOI = 0.1, 1 and 10) since it may differ between phages.
- Incubate the tubes with shaking at 37 °C for at least 2 h. Check for lysis of the phage treated cultures by visual inspection.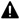 **CRITICAL STEP** Incubation should proceed until complete lysis of the culture is observed (transparent tube compared to the turbid control tube). This can take more or less time depending on the MOI, the phage and the host strain used.
- Centrifuge the lysed cultures (13,600× *g*, 15 min at 4 °C) and keep the supernatant. Titer the supernatants as indicated above.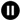 **PAUSE STEP** Phage suspensions can be kept at 4 °C for up to three months.
- Repeat the propagation protocol twice by scaling up the volume, and using the MOI resulting in the highest concentrated phage titer. Use the 10 mL phage stock obtained previously to perform propagation in 100 mL. Then, use the 100 mL phage stock to perform propagation in 1 L.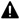 **CRITICAL STEP** The need for scaling up and the number of extra propagation rounds may vary among phages, depending on both the phage concentration obtained and the optimal MOI used for propagation.
- After carrying out propagation in 1 L volume, filter the lysate using a vacuum pump and 0.45 µm cellulose acetate vacuum filters. Concentrate the phage suspension by adding NaCl (0.5 M, final concentration) and PEG 8000 (10%, final concentration). Mix the components until they dissolve and maintain for 18 h at 4 °C. Divide the culture into four 500 mL centrifuge bottles and centrifuge at 16,000× *g* for 30 min at 4 °C.
- After centrifugation, decant the supernatant very carefully and suspend each phage pellet in 1 mL of SM buffer containing RNAse (40 µg/mL, final concentration). Measure the total volume of phage obtained.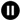 **PAUSE STEP** This phage stock can be kept at 4 °C for at least 3 months or stored at −80 °C for more than two years after adding glycerol (20% (*v*/*v*), final concentration).
- Titer the concentrated phage stock, as described previously.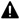 **CRITICAL STEP** The titer of the concentrated phage stock is usually 100 times higher than the one obtained in a liquid propagation.
- **OPTIONAL STEP** Perform phage purification.

### 3.4. Phage Purification. 1 Day (OPTIONAL STEP)

- Using the concentrated phage stock previously obtained, add CsCl to a final concentration of 0.75 g/mL, and mix vigorously.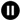 **PAUSE STEP** Mixture can be kept at 4 °C for up to 1 week.
- Transfer the mixture to two Ultra-clear centrifuge tubes. Fill the tubes with filling solution (SM buffer supplemented with 0.75 g/mL CsCl). Place the tubes into buckets and centrifuge the samples in the swinging bucket rotor at 100,000× *g*, at 4 °C for 20 h, without brake.
- After ultracentrifugation, the purified phage can be identified as a light blue band in the middle of the tube. Pinch the tube carefully and extract the band using a 2 mL syringe and a 0.5 × 16 mm needle.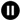 **PAUSE STEP** Purified phage stocks in a CsCl suspension can be kept at 4 °C for long periods of time (>5 years), as most phages are very stable in these conditions.

### 3.5. Sodium Pyrophosphate Treatment. 4–8 Days

**Figure 2:**
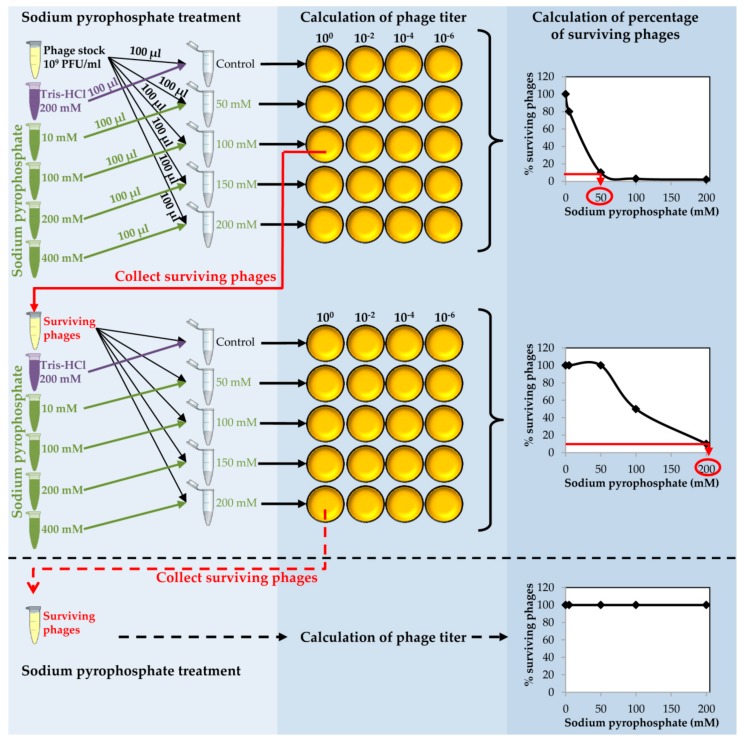
Schematic representation of the sodium pyrophosphate treatment of staphylococcal phages. Phage treatment should be repeated until phage survival reaches a plateau of 100% at every chelating agent concentration tested.

- Place 150 µL of purified phage onto 0.025 µm VSWP Membrane MF-Millipore filter floating over 10 mL of SM buffer in a Petri dish. Incubate without shaking at 20 °C for 1 h. Calculate the titer of the dialyzed phage stock.
- Take an aliquot (10 µL) of dialyzed purified phage and make a 1:100 dilution into 990 µL of SM buffer (Figure 2).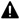 **CRITICAL STEP** The dilution step is carried out in order to obtain a phage stock of 10^9^ PFU/mL to proceed with the chelating agent treatment.
- Prepare a series of tubes with 100 µL of the diluted phage in each tube. Add 100 µL of each sodium pyrophosphate concentration (10–400 mM) to each tube in order to dilute the chelating agent 1:2 and get a final concentration from 5 to 200 mM in a final volume of 200 µL (Figure 2).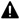 **CRITICAL STEP** For some phages, the destabilizing effect might be higher using other chelating agents such as EDTA or sodium citrate, but the procedure to follow in these cases is identical to that using sodium pyrophosphate.
- To prepare the control, mix 100 µL of purified phage with 100 µL of sterile Tris-HCl 200 mM, pH 8.
- Incubate the tubes at 37 °C for 30 min.
- Calculate the phage titer of each sample after the sodium pyrophosphate treatment making 1:100 dilutions in SM buffer and plate by the double layer technique using *S. aureus* as the host strain. At the same time, directly plate the undiluted sample (Figure 2).
- Determine the percentage of surviving phages after the treatment compared to the control sample. Results must be depicted as a function of sodium pyrophosphate concentration (Figure 2).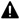 **CRITICAL STEP** The sodium pyrophosphate treatment causes a decrease in the initial phage titer that should be around 90–99%. Some phages have a very stable particle structure; in these cases, a higher concentration of the chelating agent or an increase in the incubation temperature might be necessary.
- Collect the surviving phages from the plate in which the sodium pyrophosphate treatment led to a survival rate of about 10% of the phage population (Figure 2). To do this, add 1 mL of SM buffer to the plate corresponding to the phage dilution 10° and incubate 1 h with slight shaking (60 rpm) at room temperature. Afterwards, collect the liquid and centrifuge 10 min at 13,600× *g*, at 4 °C. Keep the supernatant containing the surviving phages.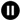 **PAUSE STEP** The phage stock can be kept at 4 °C for 1 month before continuing with the protocol.
- Repeat the sodium pyrophosphate treatment (50–200 mM), as described above using the surviving phages obtained previously (Figure 2).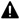 **CRITICAL STEP** Repeat the pyrophosphate treatment from 3 to 5 rounds until phage survival reaches a plateau of 100% even at high concentrations of the chelating agent (Figure 2).

### 3.6. Isolation of Phage Deletion Mutants. 1 Day

- Pick isolated lysis plaques with a sterile tip from the sample treated with the highest concentration of sodium pyrophosphate, and suspend each lysis plaque in 100 µL of SM buffer.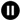 **PAUSE STEP** The phage stock can be kept at 4 °C for 1 month.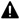 **CRITICAL STEP** The number of lysis plaques to be tested cannot be calculated in advance, but it is recommended to select about 10–20 lysis plaques for an initial screening for the desired mutant.
  **OPTIONAL STEP** Propagate the putative phage deletion mutants following previous Section 3.1 and Section 3.2.
- For temperate phages, the protocol can be extended to select virulent phages with “clear lysis plaque” phenotype (following section).

### 3.7. Selection of “Clear Lysis Plaque” Phage Deletion Mutants. 4 Days

**Figure 3:**
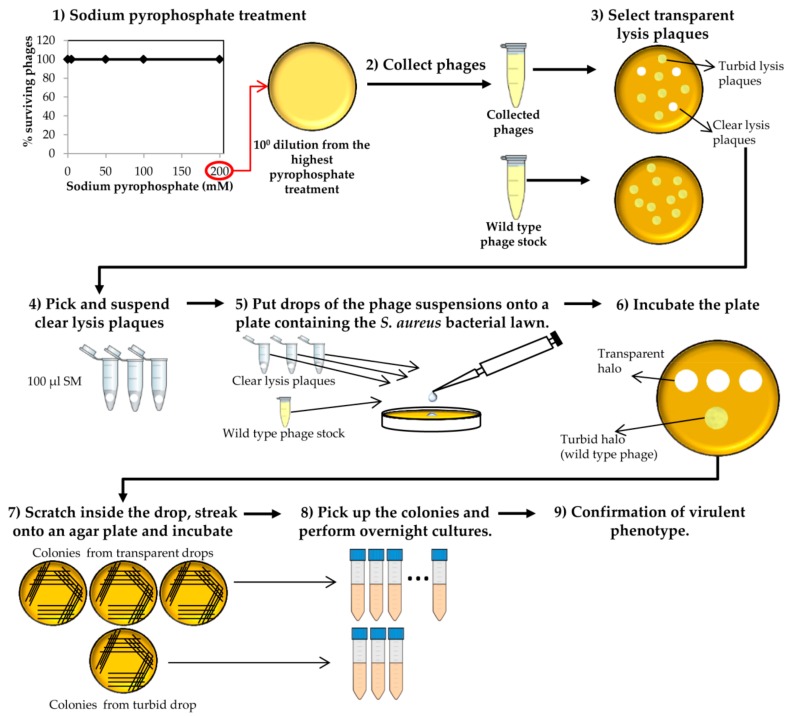
Selection of “clear lysis plaque” phage deletion mutants. This process starts with collection of the surviving phages after the last round of sodium pyrophosphate treatment.

**Figure 4:**
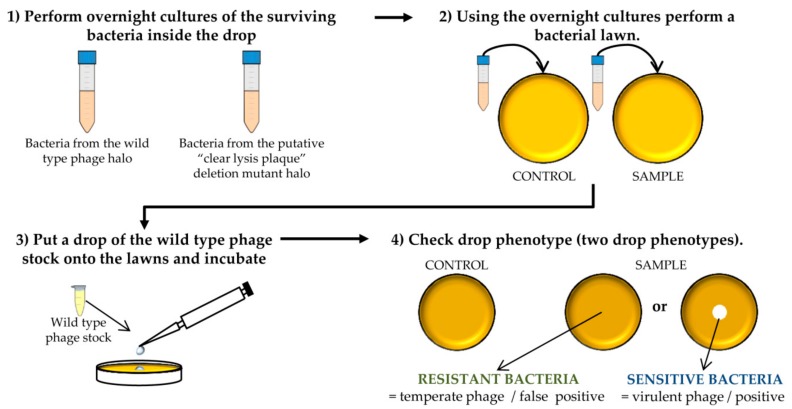
Steps performed to determine the virulent phenotype of the putative “clear lysis plaque” phage deletion mutants. This scheme represents the different experiments carried out for one putative phage mutant and the corresponding control. The protocol should be performed for all putative deletion mutants.

- Collect phages from the plate that resulted from the last round of treatment with the highest sodium pyrophosphate concentration, in which a plateau of 100% phage survival had been reached (Figure 3). To do this, add 1 mL of SM buffer to the plate corresponding to the phage dilution 10° and incubate 1 h with slight shaking (60 rpm) at room temperature. Afterwards, collect the liquid and centrifuge at 10,000 rpm for 10 min at 4 °C. Keep the supernatant containing the surviving phages.
- Make 1:10 dilutions of the phage suspensions in SM buffer and plate the appropriate dilutions using the double layer technique and *S. aureus* as host strain. For control purposes, make dilutions and plate the wild-type phage (not treated with any chelating agent). Let the medium solidify, and then incubate the plates at 37 °C for 18 h (Figure 3).
  At the same time, inoculate one isolated colony of *S. aureus* in TSB to obtain an o/n culture (see above).
- Observe carefully the lysis plaques formed by the putative phage deletion mutants and compare with those formed by the wild-type phage. Pick up each putative “clear lysis plaque” (transparent phenotype) and suspend it in 100 µL of SM buffer (Figure 3).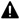 **CRITICAL STEP** For some phages, it is necessary to incubate the plates for a longer period in order to get a more evident transparent phenotype.
- Take 100 µL of the *S. aureus* o/n culture and mix with 5 mL of TSA 0.7%. Pour the mixture onto a TSA 2% plate, allowing to harden. Place a 5 µL drop of each putative phage mutant suspension and a drop of the wild-type phage stock onto the plate surface. Keep the plates at room temperature until drops become dried and then incubate at 37 °C for 18 h.
- Scratch with a sterile tip inside the halos generated by the putative “clear lysis plaque” mutants (transparent halo) and streak onto a TSA 2% plate. Repeat the same with the halo generated by the wild-type phage (turbid halo). Incubate the plates at 37 °C for 18 h (Figure 3).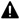 **CRITICAL STEP** The number of bacteria from the wild-type phage halo is expected to be high, while none or a low number of colonies should be present in the “clear lysis plaque” halo. Virulent phages with “clear lysis plaque” phenotype are not able to lysogenize their host; therefore, no cells are expected to survive after infection. By contrast, a high number of lysogenic cells (resistant to phage infection) will be generated inside the halo of the wild-type temperate phage.
- Confirm the lytic phenotype. Pick up 3 isolated colonies from the plate that comes from the wild-type phage halo and all the colonies from the “clear lysis plaque” mutant halo. Introduce these colonies into sterile tubes containing 3 mL TSB and incubate with shaking (250 rpm) for 18 h at 37 °C (Figure 3 and Figure 4).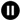 **PAUSE STEP** Prepare a stock of the colonies by adding 20% glycerol (final concentration) to the o/n cultures and freezing at −80 °C.
- Take 100 µL of each o/n culture and mix with 5 mL of TSA 0.7%. Pour the mixture onto TSA 2% plates and allow hardening. Place a 5 µL drop of the wild-type phage stock. Keep the plates at room temperature until it drops dry and then incubate them at 37 °C for 18 h (Figure 4).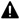 **CRITICAL STEP** After the incubation, two phenotypes can be observed: the presence of a clear halo, which means that the strain is sensitive to the phage, or complete growth of the bacteria inside the halo which indicates resistance to the phage. The presence of a transparent halo on the surviving bacterial lawn is indicative that the putative “clear lysis plaque” phage mutant is a real virulent phage.

## 4. Expected Results

The protocol described here has been used for the selection of “clear lysis plaque” phage mutants from phage phiH5 [12]. Initially, a growth curve of the host bacterium *S. aureus* Sa9 was performed at 37 °C with shaking, in order to determine the number of bacterial cells present in the exponential growing phase. The results showed that log phase started at OD_600_ = 0.1, which corresponds to 10^8^ CFU/mL. The stationary phase began after 8 h of incubation, when a total number of bacteria of ~10^9^ CFU/mL and an absorbance of ~2 were reached.

To propagate phage phiH5 in solid medium, 100 µL of an o/n culture of the host bacterium was infected with 100 µL of a phage stock with a titer of 10^7^ PFU/mL, using the double layer technique. After this first propagation step, the new phage stock (10^8^ PFU/mL) was used to repeat propagation in solid medium. In this way, the volume of phage stock was increased to 5 mL, with a concentration of 10^9^ PFU/mL.

Next, propagation in liquid medium was carried out in 5 mL of exponentially growing bacteria (OD_600 nm_ = 0.1 which corresponds to 10^8^ CFU/mL), using three different MOIs (0.1, 1 and 10). All of the cultures lysed completely after 3 h of incubation at 37 °C; however, the phage titer (10^9^ PFU/mL) obtained after infection with a MOI of 1 was higher than that obtained with the other two MOIs (10^8^ PFU/mL). Thus, a MOI of 1 seems to be optimal for the propagation of phiH5 on *S. aureus* Sa9. Then, the protocol was scaled to 50 mL, 100 mL, and 1 liter; obtaining a phage titer ~10^9^ PFU/mL after each round of propagation. Finally, the phage was purified using a CsCl gradient and dialyzed, resulting in a purified phage stock of about 10^11^ PFU/mL.

**Figure 5:**
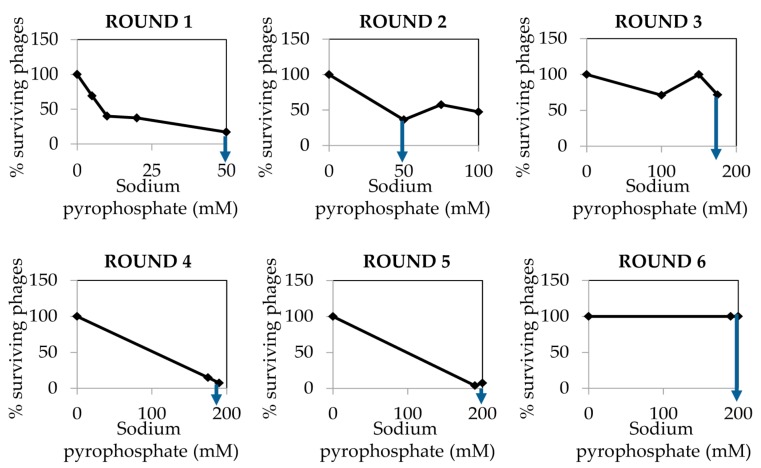
Percentage of phage phiH5 survival after treatment with sodium pyrophosphate at 37 °C for 30 min. The blue arrow indicates the treatment concentration from where phages were chosen for the next round of treatment.

The dialyzed and purified phiH5 stock was treated with increasing concentrations of sodium pyrophosphate in six different rounds, until a plateau of 100% of surviving phages was reached (Figure 5).

**Figure 6:**
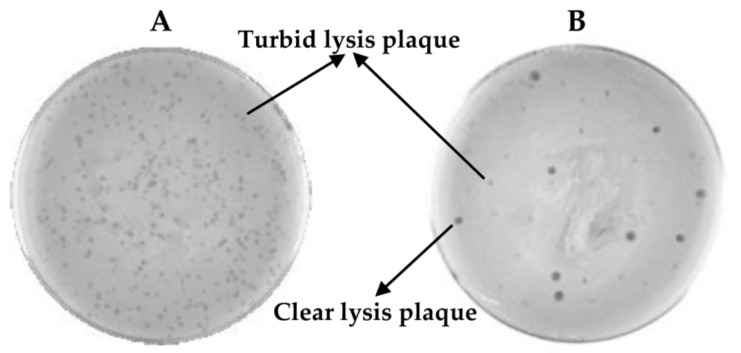
Example of phage lysis plaque phenotypes observed in (**A**) wild type phiH5 and (**B**) phiH5 collected after sodium pyrophosphate treatment onto *S. aureus* Sa9 lawn.

Once phiH5 was exposed to six rounds of pyrophosphate treatment, the surviving phages from the sixth round (sample exposed to 200 mM sodium pyrophosphate) were collected and diluted to observe isolated lysis plaques. The opacity of the resulting plaques was compared with the ones that were obtained from the wild-type phage stock. The most transparent lysis plaques (21 transparent lysis plaques, Figure 6), so-called “clear lysis plaques” were picked up and suspended in SM buffer for further analysis. A drop of each phage suspension and a drop of the wild-type phage were poured onto a *S. aureus* Sa9 lawn. After the incubation of these plates, the colonies growing inside the halo of phage lysis were scratched and streaked on agar plates to obtain single colonies. From the 21 scratched halos, only one resulted in no surviving bacteria, since no colonies were recovered. This was confirmed as a real “clear lysis plaque” phage mutant. From the remaining 20 halos and from the wild-type phage halo, three colonies were picked up to confirm their phenotype. All of these colonies resulted resistant to phage infection, indicating their lysogenic status. The putative phage mutant with “clear lysis plaque” phenotype (named phiIPLA88) was characterized by DNA restriction analysis. Since no changes in the restriction pattern were observed (data not shown), the complete genome sequence of phiIPLA88 was obtained and compared with that of the wild-type phage phiH5. A point mutation (1-base replacement) leading to the loss of the start codon was found in the repressor-encoding gene. As a consequence of this mutation, phage phiIPLA88 is an obligate virulent phage [12]. It is worth noting that mutant virulent phages can spontaneously occur in a temperate phage population, therefore, it might be possible to isolate them directly. However, the frequency of spontaneous mutation in phages is generally very low (>10^−8^) [13], and this protocol facilitates their selection.

## 5. Reagents Setup

- Medium for bacterial growth: TSB; TSA (TSB supplemented with 2% *w*/*v* agar) and semisolid TSA medium (TSB supplemented with 0.7% *w*/*v* agar). TSB is prepared following the supplier’s recommendations. For the preparation of TSA, agar should be added prior to autoclaving. Sterilize by autoclaving at 121 °C for 15 min. TSA can be stored at room temperature for up to one month. Pour TSA 2% into 9 cm plates and allowed to solidify. Plates can be kept at 4 °C for up to one month. Allow TSA 0.7% to solidify at room temperature, and then it can be stored for up to one month. Before using, melt TSA-0.7% in a microwave and tempered to 50 °C in a water bath.
- SM buffer: 200 mM Tris HCl, 10 mM MgSO_4_, 10 mM CaCl_2_, and 100 mM NaCl in distilled water. Adjust pH to 7.5 with HCl. Sterilize by autoclaving at 121 °C 15 min. The buffer can be kept for up to one month at room temperature.
- PBS buffer: 137 mM NaCl, 2.7 mM KCl, 10 mM Na_2_HPO_4_ and 2 mM KH_2_PO_4_ in distilled water. Adjust pH to 7.4 with HCl. Sterilize by autoclaving at 121 °C 15 min. The buffer can be kept for up to one month at room temperature.
- CaCl_2_ solution: dissolve CaCl_2_ into distilled water at a final concentration of 1 M. Sterilize by autoclaving at 121 °C 15 min. The solution can be kept for up to one month at room temperature.
- MgSO_4_ solution: dissolve MgSO_4_ into distilled water at a final concentration of 1 M. Sterilize by autoclaving at 121 °C 15 min. The solution can be kept for up to one month at room temperature.
- PEG 8000 solution: dissolve PEG 8000 into distilled water to a final concentration of 30% (*w*/*v*). Sterilize by autoclaving at 121 °C 15 min. The solution can be kept for up to one month at room temperature.
- NaCl solution: dissolve NaCl into distilled water to a final concentration of 5 M. Sterilize by autoclaving at 121 °C 15 min. The solution can be kept for up to one month at room temperature.
- RNAse: dissolve RNAse into double distilled water to a final concentration of 5 mg/mL (*w*/*v*). Sterilize by filtering using a 0.2 µm PES filter. The stock can be kept at −20 °C for up to the expiration date that is provided by the supplier.
- Sodium pyrophosphate solutions: dissolve sodium pyrophosphate in Tris HCl 200 mM pH = 8 at a final concentration of 400 mM and then dilute to 10 mM, 20 mM, 40 mM 100 mM, 150 mM, 200 mM, 300 mM, and 350 mM. Sterilize by autoclaving at 121 °C 15 min. These solutions can be kept for up to one month at room temperature.
- Tris HCl 100 mM pH = 7.4: dissolve Tris HCl into distilled water to a final concentration of 100 mM. Adjust pH to 7.4 with HCl. Sterilize by autoclaving at 121 °C 15 min. The solution can be kept for up to one month at room temperature.
